# Chlamydia-related knowledge, opinion to opportunistic testing, and practices of providers among different sexually transmitted infections related departments in hospitals in Shenzhen city, China

**DOI:** 10.1186/s12913-022-08012-3

**Published:** 2022-05-04

**Authors:** Rongxing Weng, Chunlai Zhang, Lizhang Wen, Yiting Luo, Jianbin Ye, Honglin Wang, Jing Li, Ning Ning, Junxin Huang, Xiangsheng Chen, Yumao Cai

**Affiliations:** 1 Department of STD Control and Prevention, Shenzhen Center for Chronic Disease Control, No. 2021, Buxin Road, Luohu District, Shenzhen City, Guangdong Province China; 2 Shantou University Medical College, Shantou, 515000 China; 3 Chinese Academy of Medical Sciences & Peking Union Medical College Institute of Dermatology, Nanjing, China; 4 National Center for STD Control, China Center for Disease Control and Prevention, Nanjing, China

**Keywords:** *Chlamydia trachomatis*, Providers, Opportunistic testing

## Abstract

**Background:**

*Chlamydia trachomatis* (CT) infection could lead to seriously adverse outcomes if left untreated. This study aimed to determine CT-related knowledge, opinion to testing, and practices of providers among different sexually transmitted infections (STI) related departments in hospitals in Shenzhen city, China, and also to explore the differences in these responses.

**Materials and methods:**

From 1st April 2018 to 15th April 2018, a cross-sectional study was conducted in Shenzhen and 64 of 66 hospitals agreed to participate in this study. In the hospital sites, all the providers from the department of obstetrics and gynecology, department of dermatology and venereology, department of urology, and anorectal surgical department were recruited. A structured paper-based questionnaire was used to obtain data on CT-related information.

**Results:**

A total of 355 providers from 64 hospitals participated in the current study. Compared to providers from the department of dermatology and venereology, those from the department of obstetrics and gynecology (OR = 0.31, 95% CI 0.16—0.62), department of urology (OR = 0.32, 95% CI 0.16—0.65), and anorectal surgical department (OR = 0.25, 95% CI 0.09—0.71) were less likely to identify that “Be in a long-term mutually monogamous relationship with a partner who has been tested and has negative STI test results.” is an appropriate way for a sexually active person to reduce risk of getting CT. Also, those from the department of obstetrics and gynecology (OR = 0.45, 95% CI 0.23—0.87) were less likely to identify that “Use latex condoms the right way every time you have sex” is another appropriate way. A high proportion of providers agreed that all sexually active patients attending to their department should be screened regularly (77.1%), and they are willing to offer opportunistic CT screening (96.0%). Only 11.4% of respondents correctly identified that the appropriate time frame of the CT retesting is three months.

**Conclusions:**

Providers among STI-related departments in hospitals showed a very high willingness to offer opportunistic CT screening. However, this study showed important gaps in providers’ knowledge and practices in China, targeted training in CT-related knowledge and practice is urgently needed.

**Supplementary Information:**

The online version contains supplementary material available at 10.1186/s12913-022-08012-3.

## Background

*Chlamydia trachomatis* (CT) infection in the genital area is one of the most common sexually transmitted infections (STI) throughout the world. According to the World Health Organization (WHO), the global prevalence of CT infection in men and women aged 15–49 years in 2016 was 2.7% and 3.8%, with the Western Pacific Region ranking the top among all the WHO regions [1]. In China, the incidence of CT infection was 72.2/100,000 in 2019 and the burden of CT infection in Shenzhen city was much greater (249.4/100,000 in 2019), which was also the largest among cities in Guangdong province [2, 3]. Untreated CT infection in women may lead to serious complications, including pelvic inflammatory disease (PID), ectopic pregnancy, tubal infertility, and chronic pelvic pain [4, 5]. CT infection in pregnant women contributes to the incidence of adverse obstetric outcomes such as preterm birth and low birthweight [6]. Among men, CT infection is associated with non-gonococcal urethritis, epididymitis, and infertility [7, 8]. Moreover, genital CT infection significantly increases the risk of HIV infection and human papillomavirus (HPV)-associated cervical carcinoma development [9–11]. Approximately 85% of CT infection in women and men are asymptomatic [12], thus the majority of infection would have been missed if screening only those with clinical symptoms. In China, no routine CT screening is required by the national guidelines, and physicians mainly offer CT tests to those with clinical symptoms, leading to a missed opportunity to identify asymptomatic CT infection. Missed asymptomatic CT infection screening is a missed public health opportunity to prevent further transmission through detection and treatment.

Previous studies reported various risk factors of CT infection that may help identify an at-risk population for CT screening. A previous study in Jiangsu province, China showed that, for women attending STI and gynecology clinics, those who were service personnel or farmers, lived in South Jiangsu, and were from STI clinics were more likely to infect CT [13]. A study in Guangdong province, China indicated that, for pregnant women, gynecology clinic attendees, and subfertile women, risk factors of CT infection included first intercourse before 25 years of age, with multipara, and ever having more than 1 partner [14]. A previous study in Shenzhen city, China reported that, for patients attending sexual and reproductive health clinics, those who were 18–25 years old, never tested for CT before, and infected with NG were more likely to infect CT [15]. In addition, a study in Shenzhen city reported that, for asymptomatic men attending to sexually transmitted disease related clinics, less than 30 years old, being employed in the commercial service work, and being recruited through the urological department were risk factors of CT infection, and for asymptomatic women, less than 30 years old was a risk factor of CT infection [16]. Also, for the general population, a study in Shandong province, China found that, for men, first intercourse before 20 years of age and having two or more lifetime sex partners were risk factors of CT infection, and for women, being unmarried, divorced, or widowed and having two or more lifetime sex partners were risk factors of CT infection [17].

Opportunistic CT screening was recommended and applied in many projects to expand the detection of CT cases [18–20]. Similarly, the Shenzhen Gonorrhea and Chlamydia Intervention Programme (SGCIP) [21], a pilot project of a national programme in China (the China Chlamydia Intervention Programme), also aimed to promote opportunistic CT screening in China, and providers in STI-related departments in hospitals will play a major role to perform opportunistic CT tests. Generally, in China, patients with STI-related symptoms are likely to seek consultation and treatment in several outpatient departments in hospitals including the department of obstetrics and gynecology, department of dermatology and venereology, department of urology, and anorectal surgical department [16, 22]. Several standardized education materials published globally and nationally could be used to inform providers about the management and treatment of CT infection [23–25]. In China, the National Sexually Transmitted Disease Treatment Guidelines (National STD Guidelines) were provided for all the providers to obtain STI-related information including CT diagnosis and treatment, CT-related knowledge, retesting, and partner notification [25].

However, little is known about the CT-related knowledge, opinion to testing, and practices of providers in the Chinese context. Although providers in the department of dermatology and venereology are well trained during their education, providers in other departments are also involved in a large amount of CT tests and cases reports [26, 27]. Therefore, having a basic understanding of the CT-related knowledge, opinion to opportunistic testing, and practices of providers in different departments could inform the design of SGCIP interventions for the promotion of opportunistic CT screening. This study aimed to 1) determine CT-related knowledge, opinion to opportunistic testing, and practices of providers among different STI-related departments in hospitals in Shenzhen city, China; 2) to explore the differences in the responses between providers from the department of dermatology and venereology and providers from the department of obstetrics and gynecology, department of urology, and anorectal surgical department; 3) to explore associated factors of the willingness to offer opportunistic CT screening.

## Methods

### Setting and participants

This study belonged to a part of a baseline study in the Shenzhen Gonorrhea and Chlamydia Intervention Programme. There were 66 district-level or above hospitals in Shenzhen city in 2018, which were selected in the study. Totally, 64 of 66 hospitals agreed to participate in this study. In the hospital sites, all the providers from the department of obstetrics and gynecology, department of dermatology and venereology, department of urology, and anorectal surgical department were eligible and recruited. From 1st April 2018 to 15th April 2018, all the providers were invited to fill out a questionnaire after signing a written informed consent.

### Questionnaire

The questionnaire survey was led by trained staff at each study site. A structured paper-based, self-administered questionnaire was used to obtain CT-related information, with using several types of questions including Yes or No, multiple-choice questions, and text responses. This questionnaire consists of five sections including Basic information (Sect. 1: two questions), CT-related knowledge (Sect. 2: four questions), provider opinion to CT testing (Sect. 3: three questions), Training and CT testing experience (Sect. 4: five questions), CT practice (Sect. 5: four questions including retesting and partner notification, and treatment). For participants who reported involvement in CT testing, they were also asked to complete continued questions in Sect. 4 including the amounts of CT tests they offered to patients per month, CT testing choice, and specimen collection. This questionnaire was designed to have a basic understanding of providers’ knowledge about CT infection, opinion to CT testing, and clinical practice. The development of questions in Sect. 2 was based on the information from the centers for disease control and prevention in the United States (US) [28]. Potential important opinions in Sect. 3 were designed based on previous findings in the SGCIP. The development of questions in Sects. 4 and 5 was mainly based on the information in National STD Guidelines. The average time for finishing the questionnaire was around 5 min.

### Explanation of the questions in the questionnaire

The term ‘all sexually active patients’ in the question of ‘All sexually active patients attending to your department should be screened regularly’ included patients with a lower risk of CT infection such as those with monogamous relationships, and patients with a higher risk of CT infection such as those having a new sexual partner or multiple sex partners. The correct answer to the question ‘Offering opportunistic CT testing would cause an economic burden to patients’ was ‘No’, as CT testing was covered under medical insurance in China. The correct answer of the appropriate frame of CT retesting after treatment was three months. The correct answers of the first choice of treatment for men or non-pregnant women with CT infection were azithromycin or doxycycline, and the first choice of treatment for pregnant women with CT infection was azithromycin.

### Theory of Planned Behaviour (TPB) questions for willingness to offer opportunistic CT screening

The questions of ‘All sexually active patients attending to your department should be screened regularly’, ‘Is opportunistic CT screening an effective way to detect more CT infections?’, and ‘Offering opportunistic CT testing would cause an economic burden to patients’ were considered as the attitudes for willingness to offer opportunistic CT screening.

### Statistical analysis

All data from the questionnaires and laboratory results were double entered into the computer using Epi Data software (Epi Data for Windows; The Epi Data Association Odense, Denmark) to establish a dataset. Frequencies (%) were used in categorical variables. All the variables were included in the multinomial logistic regression to explore the differences in the responses between providers from the department of dermatology and venereology and providers from the department of obstetrics and gynecology, department of urology, and anorectal surgical department, except for the response of treatment for pregnant women due to a large proportion of missing data (34.3%) and the continued questions for participants who reported involvement in CT testing. Univariate logistic regression analysis was used to examine the association between TPB variables and willingness to offer opportunistic CT screening and TPB variables with *P* < 0.1 were included in multivariate logistic regression analysis using a forward stepwise procedure to obtain adjusted odds ratios (AOR) and their 95% CIs. All data analysis was performed with Statistical Package for Social Sciences (SPSS) version 21.0 software (SPSS Inc., Chicago, IL, USA). All tests were two-tailed, and *P* < 0.05 was defined as statistical significance.

## Results

### Training and CT testing experience

A total of 355 providers from 64 hospitals participated in the current study. As shown in Table 1, most of the participants (89.1%) have attended to the training on STIs diagnosis and treatment, and around three-quarters of them (72.9%) reported involvement in CT testing. Among those who reported involvement in CT testing, a similar proportion of them reported that they were performing between 1 – 5 (42.6%) and between 6 – 10 (40.3%) CT tests per month.

**Table 1:** Training and CT testing experience

Variables	Overall n (%)	D&V Dept n (%)	Ob/Gyn Dept n (%)	OR (95% CI)	*P* Values^a^	Urology Dept n (%)	OR (95% CI)	*P* Values^b^	Anorectal surgical Dept n (%)	OR (95% CI)	*P* Values^c^
Training of STIs diagnosis and treatment. (*n* = 351)											
Yes	313 (89.2)	98 (93.3)	108 (92.3)	Reference		73 (86.9)	Reference		34 (75.6)	Reference	
No	38 (10.8)	7 (6.7)	9 (7.7)	1.43 (0.42,4.85)	0.567	11 (13.1)	2.06 (0.61,7.01)	0.246	11 (24.4)	1.36 (0.22,8.26)	0.737
Involvement in CT testing in last 3 months (*n* = 354)											
Yes	258 (72.9)	80 (75.5)	91 (77.8)	0.93 (0.42,2.03)	0.849	68 (80.0)	1.70 (0.71,4.08)	0.233	19 (41.3)	0.68 (0.21,2.22)	0.518
No	96 (27.1)	26 (24.5)	26 (22.2)	Reference		17 (20.0)	Reference		27 (58.7)	Reference	

Abbreviations: *CI* confidence interval, *CT* chlamydia trachomatis, *Dept.* department, *D&V* dermatology and venereology, *Ob/Gyn* obstetrics and gynecology, *OR* odds ratio, *STI* sexually transmitted infections

^*^P < 0.05

^a^*P* values between D&V Dept. and Ob/Gyn Dept

^b^*P* values between D&V Dept. and Urology Dept

^c^*P* values between D&V Dept. and Anorectal surgical Dept

### CT testing choice and specimen collection

Those who reported involvement in CT testing were also asked questions about testing choice and specimen collection. Around half of them (51.8%) indicated that they would choose the nucleic acid amplification test (NAAT) as their first choice to detect CT infection and 70% of them answered that extragenital sites (oral or rectal swab) should be considered for men who have sex with men.

### CT related knowledge

Most providers (82.5%) correctly answered that CT is a common sexually transmitted infection (see Table 2). Compared to providers from the department of dermatology and venereology, those from the department of obstetrics and gynecology (OR = 0.31, 95% CI 0.16—0.62), department of urology (OR = 0.32, 95% CI 0.16—0.65), and anorectal surgical department (OR = 0.25, 95% CI 0.09—0.71) were less likely to identify that “Be in a long-term mutually monogamous relationship with a partner who has been tested and has negative STI test results.” is an appropriate way for a sexually active person to reduce risk of getting CT (Table 1). Also, compared to providers from the department of dermatology and venereology, those from the department of obstetrics and gynecology (OR = 0.45, 95% CI 0.23—0.87) were less likely to identify that “Use latex condoms the right way every time you have sex” is another appropriate way for a sexually active person to reduce risk of getting chlamydia (Table 2). Near one-third of respondents (64.4%) believed that opportunistic CT screening is an effective way to detect more CT infections, and compared to providers from the department of dermatology and venereology, those from the anorectal surgical department (OR = 3.75, 95% CI 1.01 – 13.89) were more likely to choose “Yes” in this question (Table 2).

**Table 2:** CT-related knowledge

Variables	Overall n (%)	D&V Dept n (%)	Ob/Gyn Dept n (%)	OR (95% CI)	*P* Values^a^	Urology Dept n (%)	OR (95% CI)	*P* Values^b^	Anorectal surgical Dept n (%)	OR (95% CI)	*P* Values^c^
CT is a common sexually transmitted infection. (*n* = 355)											
Yes	293 (82.5)	90 (84.9)	94 (79.7)	0.58 (0.25,1.31)	0.187	70 (82.4)	0.75 (0.31,1.82)	0.524	39 (84.8)	1.05 (0.28,3.96)	0.947
No	62 (17.5)	16 (15.1)	24 (20.3)	Reference		15 (17.6)	Reference		7 (15.2)	Reference	
How can a sexually active person reduce the risk of getting chlamydia?											
1. Be in a long-term mutually monogamous relationship with a partner who has been tested and has negative STI test results. (*n* = 354)											
Yes	199 (56.2)	75 (70.8)	61 (52.1)	0.31 (0.16,0.62)	0.001^*^	40 (47.1)	0.32 (0.16,0.65)	0.002^*^	23 (50.0)	0.25 (0.09,0.71)	0.010^*^
No	155 (43.8)	31 (29.2)	56 (47.9)	Reference		45 (52.9)	Reference		23 (50.0)	Reference	
2. Use latex condoms the right way every time you have sex. (*n* = 354)											
Yes	210 (59.3)	76 (71.7)	64 (54.7)	0.45 (0.23,0.87)	0.017^*^	45 (52.9)	0.50 (0.25,1.00)	0.051	25 (54.3)	0.84 (0.29,2.46)	0.751
No	144 (40.7)	30 (28.3)	53 (45.3)	Reference		40 (47.1)	Reference		21 (45.7)	Reference	
Opportunistic CT screening is an effective way to detect more CT infections. (*n* = 354)											
Yes	228 (64.4)	67 (63.2)	71 (60.7)	1.09 (0.55,2.15)	0.800	54 (63.5)	1.05 (0.51,2.14)	0.904	36 (78.3)	3.75 (1.01,13.89)	0.047^*^
No	126 (35.6)	39 (36.8)	46 (39.3)	Reference		31 (36.5)	Reference		10 (21.7)	Reference	

^*^P < 0.05

Abbreviations: *CI* confidence interval, *CT* chlamydia trachomatis, *Dept.* department, *D&V* dermatology and venereology, *Ob/Gyn* obstetrics and gynecology, *OR* odds ratio, *STI* sexually transmitted infections

^a^*P* values between D&V Dept. and Ob/Gyn Dept

^b^*P* values between D&V Dept. and Urology Dept

^c^*P* values between D&V Dept. and Anorectal surgical Dept

### Provider opinion to testing

As shown in Table 3, a high proportion of providers agreed that all sexually active patients attending to their department should be screened regularly (77.1%), they are willing to offer opportunistic CT screening (96.0%), and offering opportunistic CT testing would not cause economic burden to patients (89.2%).

**Table 3:** Provider opinion to testing

Variables	Overall n (%)	D&V Dept n (%)	Ob/Gyn Dept n (%)	OR (95% CI)	*P* Values^a^	Urology Dept n (%)	OR (95% CI)	*P* Values^b^	Anorectal surgical Dept n (%)	OR (95% CI)	*P* Values^c^
All sexually active patients attending to your department should be screened regularly. (n = 354)											
Yes	273 (77.1)	84 (79.2)	93 (79.5)	0.93 (0.43,2.02)	0.862	57 (67.1)	0.55 (0.25,1.20)	0.134	39 (84.8)	1.31 (0.33,5.30)	0.701
No	81 (22.9)	22 (20.8)	24 (20.5)	Reference		28 (32.9)	Reference		7 (15.2)	Reference	
Offering opportunistic CT testing would cause an economic burden to patients. (n = 353)											
No	315 (89.2)	95 (90.5)	107 (90.7)	1.92 (0.63,5.82)	0.248	73 (86.9)	1.41 (0.45,4.38)	0.555	40 (87.0)	0.70 (0.18,2.75)	0.608
Yes	38 (10.8)	10 (9.5)	11 (9.3)	Reference		11 (13.1)	Reference		6 (13.0)	Reference	
Willingness to offer opportunistic CT screening (n = 354)											
Willing	340 (96.0)	104 (98.1)	113 (96.6)	Reference		80 (94.1)	Reference		43 (93.5)	Reference	
Unwilling	14 (4.0)	2 (1.9)	4 (3.4)	1.49 (0.21,10.87)	0.692	5 (5.9)	2.11 (0.31,14.48)	0.446	3 (6.5)	3.03 (0.29,32.18)	0.357

Abbreviations: *CI* confidence interval, *CT* chlamydia trachomatis, *Dept.* department, *D&V* dermatology and venereology, *Ob/Gyn* obstetrics and gynecology, *OR* odds ratio

^a^*P* values between D&V Dept. and Ob/Gyn Dept

^b^*P* values between D&V Dept. and Urology Dept

^c^*P* values between D&V Dept. and Anorectal surgical Dept

### CT practice

#### Retesting and partner notification

Only 11.4% of respondents correctly identified that the appropriate time frame of the CT retesting is three months, and compared to providers from the department of dermatology and venereology, those from the department of obstetrics and gynecology (OR = 3.66, 95% CI 1.32 – 10.10) were more likely to correctly answer this question (see Table 4). Most providers (90.8%) in this study were willing to remind CT positive patients of partner notification, and compared to providers from the department of dermatology and venereology, those from the department of obstetrics and gynecology (OR = 0.21, 95% CI 0.06 – 0.69) showed less willingness on this question (see Table 4).

**Table 4:** CT practice: Retesting and partner notification

Variables	Overall n (%)	D&V Dept n (%)	Ob/Gyn Dept n (%)	OR (95% CI)	*P* Values^a^	Urology Dept n (%)	OR (95% CI)	*P* Values^b^	Anorectal surgical Dept n (%)	OR (95% CI)	*P* Values^c^
Willingness to remind CT-positive patients of partner notification. (n = 326)											
Willing	296 (90.8)	95 (95.0)	100 (87.0)	0.21 (0.06,0.69)	0.010^*^	73 (90.1)	0.32 (0.09,1.20)	0.092	28 (93.3)	0.82 (0.08,8.01)	0.864
Unwilling	30 (9.2)	5 (5.0)	15 (13.0)	Reference		8 (9.9)	Reference		2 (6.7)	Reference	
Retesting after treatment (*n* = 324)											
3 months	37 (11.4)	6 (6.0)	21 (18.6)	3.66 (1.32,10.10)	0.012^*^	8 (9.9)	1.95 (0.61,6.21)	0.260	2 (6.7)	1.21 (0.21,6.91)	0.827
Others	287 (88.6)	94 (94.0)	92 (81.4)	Reference		73 (90.1)	Reference		28 (93.3)	Reference	

Abbreviations: *CI* confidence interval, *CT* chlamydia trachomatis, *Dept.* department, *D&V* dermatology and venereology, *Ob/Gyn* obstetrics and gynecology, *OR* odds ratio

^*^P < 0.05

^a^*P* values between D&V Dept. and Ob/Gyn Dept

^b^*P* values between D&V Dept. and Urology Dept

^c^*P* values between D&V Dept. and Anorectal surgical Dept

#### Treatment

Most providers in the current study correctly chose azithromycin (53.9%) or doxycycline (32.4%) as their first choice when they prescribe for men or non-pregnant women (see Table 5). For pregnant women with uncomplicated chlamydia infection, about two-thirds of them chose azithromycin (64.1%) as their first choice when they prescribe for pregnant women, with 21.4% choosing erythromycin.

**Table 5:** CT practice: Treatment

Variables	Overall n (%)	D&V Dept n (%)	Ob/Gyn Dept n (%)	OR (95% CI)	*P* Values^a^	Urology Dept n (%)	OR (95% CI)	*P* Values^b^	Anorectal surgical Dept n (%)	OR (95% CI)	*P* Values^c^
Treatment for men or non-pregnant women (*n* = 306)											
Azithromycin	165 (53.9)	49 (51.6)	57 (52.3)	Reference		46 (61.3)	Reference		13 (48.1)	Reference	
Doxycycline	99 (32.4)	30 (31.6)	41 (37.6)	1.76 (0.87,3.55)	0.117	18 (24.0)	0.91 (0.42,1.99)	0.812	10 (37.0)	2.04 (0.69,6.01)	0.195
Others	42 (13.7)	16 (16.8)	11 (10.1)	0.66 (0.26,1.69)	0.383	11 (14.7)	0.81 (0.32,2.07)	0.658	4 (14.8)	0.83 (0.19,3.61)	0.803

Abbreviations: *CI* confidence interval, *CT* chlamydia trachomatis, *Dept.* department, *D&V* dermatology and venereology, *Ob/Gyn* obstetrics and gynecology, *OR* odds ratio

^a^*P* values between D&V Dept. and Ob/Gyn Dept

^b^*P* values between D&V Dept. and Urology Dept

^c^*P* values between D&V Dept. and Anorectal surgical Dept

#### Association between TPB variables and willingness to offer opportunistic CT screening

Results from the multivariate logistic regression model suggested that participants who agreed that “Offering opportunistic CT testing would cause an economic burden to patients” (AOR = 0.05, 95% CI = 0.02–0.16) were less willing to offer opportunistic CT screening than those who did not agree (Table 6).

**Table 6:** Multivariable logistic regression analysis of factors associated with willingness to offer opportunistic CT screening among providers

Variables	Crude OR (95% CI)	*P* Values	Adjusted OR (95% CI)	*P* Values
Attitude				
Opportunistic CT screening is an effective way to detect more CT infections. (*n* = 354)				
Yes	1.86 (0.64, 5.42)	0.257		
No	Reference			
All sexually active patients attending to your department should be screened regularly. (*n* = 354)				
Yes	2.65 (0.89, 7.87)	0.079	3.09 (0.93, 10.26)	0.065
No	Reference		Reference	
Offering opportunistic CT testing would cause an economic burden to patients. (*n* = 353)				
No	Reference		Reference	
Yes	0.05 (0.02, 0.17)	< 0.001^**^	0.05 (0.02, 0.16)	< 0.001^**^

Abbreviations: *CI* confidence interval, *CT* chlamydia trachomatis, *OR* odds ratio

^**^P < 0.001

## Discussion

To our knowledge, this is the first study to investigate CT-related knowledge, opinion to opportunistic testing, and practices of providers in China and it revealed important gaps in providers’ knowledge and practices in China, which may lead to low CT testing rates among patients attending to these STI-related departments.

Although most providers reported that they have attended to the training on STIs diagnosis and treatment, there were still around a quarter of participants who have not been involved in CT testing in the past three months. Also, a high proportion of participants have insufficient knowledge in ways for the general population to reduce the risk of getting chlamydia and opportunistic CT screening. Compared to providers from other departments, providers from the department of dermatology and venereology were more likely to identify that “Be in a long-term mutually monogamous relationship with a partner who has been tested and has negative STI test results.” is an appropriate way for a sexually active person to reduce risk of getting CT. Similarly, providers from the department of dermatology and venereology, compared to providers from the department of obstetrics and gynecology, were more likely to identify that “Use latex condoms the right way every time you have sex” is another appropriate way for a sexually active person to reduce risk of getting chlamydia. This implied that targeted education about CT-related knowledge should be delivered to all providers, especially for those from the department of obstetrics and gynecology. CT-related knowledge is very important for providers to make a significant impact on CT screening [29], and continuing medical education was recognized as a facilitator to increase CT testing [30]. The prevalence of CT infection in other departments was also high in China [16, 31] and a previous study found that reported CT cases from the department of dermatology and venereology made up only 20% of total CT cases [32]. The results of the current study indicate that addressing the differences in CT-related knowledge between departments is very important to promote opportunistic CT screening for eligible patients.

Providers reported a high willingness to offer opportunistic CT screening (96%), and most of them agreed that offering opportunistic CT testing would not cause an economic burden to patients, which indicated that promoting opportunistic CT screening in these departments is acceptable among providers. However, how to translate the high willingness to actual CT testing should also be considered. It is noticed that there are several facilitators to increase CT testing such as enhancing communication skills [33], updating knowledge about the procedure for contact tracing, training on the benefits of screening, and raising awareness of screening [34]. Also, a high proportion of providers in this study agreed that all sexually active patients attending to their department should be screened regularly, which showed a greater commitment to opportunistic CT screening. However, ignorance of patients’ age may lead to over-testing in older age groups with a lower risk of CT infection. A previous study in China showed that the CT prevalence among community women and patients attending sexual and reproductive health clinics with higher ages (25 – 49 years) were also high [15, 35], which may be the reason why providers were willing to offer CT testing to all sexually active patients. In the future CT screening promotion, the government should consider the recommended age groups in the Chinese context to ensure people with the highest risk could be all tested and achieve high cost-effectiveness.

Many countries have implemented opportunistic CT screening routinely such as the United Kingdom (UK), Australia, Sweden, and Denmark [36, 37]. The National Chlamydia Screening Programme (NCSP) in the UK was initiated in 2003 and the updated opportunistic CT screening strategy was to focus on all sexually active women under the age of 25 [38]. One of the rationales of this programme was that women with a CT screen had a lower risk of development of PID compared to those without CT screening [39]. Previous studies reported benefits of the NCSP, including a consistent decline in the average duration of CT infections concurrent with large-scale of CT testing and the cost-effectiveness of opportunistic CT screening [38, 40]. Also, annual opportunistic screening for chlamydia in women under 25 was considered as a cost-effective strategy in both the US and Australia [41, 42]. Although there were no such national strategies for implementing opportunistic screening in China, in the future, opportunistic CT screening could be considered to be integrated with some successful STI screening strategies such as early screening for syphilis among pregnant women, which was introduced in 2002 in China [43]. Also, the results from the current study could help inform the health authorities in China to develop related strategies.

In CT practice, for providers with involvement in CT testing in the past three months, only half of them would choose NAAT as their first choice to detect CT infection. This is likely due to the fact that a large proportion (54.4%) of sexually transmitted disease laboratories in Shenzhen city only used immune chromatographic-rapid diagnostic tests to detect CT infection, which could lead to missed positive CT cases [44]. A previous study in Guangdong province showed that the testing capability of sexually transmitted disease laboratories was much poorer at the province level, with only 14.53% of STD laboratories using NAATs to detect CT infection [45]. Therefore, providers may choose CT testing based on the capability of their institute, and it is important for hospitals to improve the capability to perform high sensitivity and specificity tests (e.g. NAAT) before promoting opportunistic CT screening. During the COVID-19 pandemic in China, anyone with a fever seeking healthcare in secondary and tertiary hospitals should be screened by polymerase chain reaction (PCR) testing, and the capability of laboratories in these hospitals was improved [46], so further investigation of the responses of this question along with the testing capability of their institute is required for the above interpretation. Only around 10% of respondents correctly chose the appropriate frame of CT retesting, which was much lower than the general practitioners in Australia (66%) [47]. In the (National STD Guidelines) [25], it recommends that patients need to take a repeat CT testing at 3 months after being diagnosed with CT infection. A short time frame (2 weeks to 4 weeks) may lead to a false-positive result because of the presence of non-viable chlamydial DNA [48]. Also, a considerable of providers (21.4%) selected erythromycin as their first choice when they prescribe for pregnant women with uncomplicated chlamydia infection, which is also inconsistent with the guidelines. Erythromycin could lead to more gastrointestinal side effects in pregnancy [49, 50] and frequent use of erythromycin could induce hepatotoxicity [51], so it is not recommended in the treatment of pregnant CT cases. The unfamiliarity with guidelines for diagnosis and treatment of sexually transmitted diseases suggests that training or education about the CT retesting timeframe is urgently needed for providers in these departments. A previous study in China reported that appropriate programmes to train physicians in China on STI knowledge, diagnosis, and treatment could effectively reduce the patients’ STI risk [52].

The current study reported that the cost of CT screening was a significant factor associated with the willingness to offer opportunistic CT screening. Previous studies found that the cost of a STI/HIV testing was also considered as a negative attitude towards STI/HIV testing [53, 54]. However, offering opportunistic CT testing would not cause an economic burden to patients, as CT testing was covered under medical insurance in China. Results suggested that this negative attitude towards offering opportunistic CT screening could be potentially changed with the demonstration of medical insurance coverage for CT screening. This study mainly focused on the attitudes towards offering opportunistic CT testing, other elements of TPB such as injunctive norm, descriptive norm, moral norm, and perceived behavioural control should be further explored in future studies to identify more barriers and facilitators of offering opportunistic CT testing.

Some limitations in the current study should be highlighted. First, convenient sampling was used to recruit participants, which might have limited the representativeness of our study. However, with almost all the district-level or above hospitals in Shenzhen city participating in the study (64/66), a certain level of representativeness is achieved. Second, the main limitation was that the response rate of the study was not obtained and this issue should be considered in future studies in this field. Third, self-report bias may exist in the questionnaire survey. For example, there was no independent data source to verify self-reported data such as the number of CT tests that providers performed per month and the type of test that they would choose as their first choice to detect CT infection. Last, information about the identification of patients who need STI testing was not obtained in the study. Related questions in the following “1) How to identify sexually active patients or those engaging in risk behaviours? 2) Are providers confident to ask about patients’ risk behaviours?” should be further considered in future studies.

## Conclusions

Playing a major role in CT screening promotion, providers among STI-related departments in hospitals showed a very high willingness to offer opportunistic CT screening. However, this study showed important gaps in providers’ knowledge and practices in China, targeted training in CT-related knowledge and practice is urgently needed. Also, the cost of CT screening was identified as the barrier of promoting opportunistic CT testing among providers, and the demonstration of medical insurance coverage for CT screening should be delivered to them.

## Supplementary Information

**Additional file 1.** Questionnaire for providers on the knowledge, opinions, and practice of Chlamydia trachomatis.

## Data Availability

The datasets used and/or analysed during the current study are available from the corresponding author on reasonable request. Data request should be submitted to Dr. Yumao Cai (64165469@qq.com) who will review the data request with the Ethical Review Committee of Shenzhen Center for Chronic Disease Control for approval.

## Abbreviations

CI: Confidence Interval
CT: Chlamydia Trachomatis
Dept.: Department
D&V: Dermatology and Venereology
HPV: Human Papillomavirus
NAAT: Nucleic Acid Amplification Test
Ob/Gyn: Obstetrics and Gynecology
OR: Odds Ratios
PID: Pelvic Inflammatory Disease
SD: Standard Deviation
SGCIP: Shenzhen Gonorrhea and Chlamydia Intervention Programme
SPSS: Statistical Package for Social Sciences
STI: Sexually Transmitted Infection
TPB: Theory of Planned Behaviour
WHO: World Health Organization
