# Supplementary material for: Chlamydia-related knowledge, opinion to opportunistic testing, and practices of providers among different sexually transmitted infections related departments in hospitals in Shenzhen city, China
Source: BMC Health Serv Res. 2022 May 4;22:601. doi: 10.1186/s12913-022-08012-3 (PMC9067339; doi:10.1186/s12913-022-08012-3)
Supplement: Supplementary file 1 — Additional file 1. Questionnaire for providers on the knowledge, opinions, and practice of Chlamydia trachomatis. [file 12913_2022_8012_MOESM1_ESM.docx]

**Questionnaire for providers on the knowledge, opinions, and practice of Chlamydia trachomatis**

1. **Basic Information**
   1. Name of the hospital: _____________
   2. Department:

➀ department of dermatology and venereology; ➁ department of obstetrics and gynecology; ➂ department of urology; ➃ anorectal surgical department

1. **Chlamydia trachomatis related knowledge**

2.1. Is Chlamydia trachomatis a common sexually transmitted infection?

➀Yes ➁No

2.2. How can a sexually active person reduce the risk of getting chlamydia?

a. Be in a long-term mutually monogamous relationship with a partner who has been tested and has negative STI test results.

➀Yes ➁No

b. Use latex condoms the right way every time you have sex.

➀Yes ➁No

2.3. Is opportunistic CT screening an effective way to detect more CT infections?

➀Yes ➁No

1. **Opinion to Chlamydia trachomatis testing**

3.1. Do you think that all sexually active patients attending to your department should be screened regularly?

➀Yes ➁No

3.2. Do you think that offering opportunistic CT testing would cause an economic burden to patients?

➀Yes ➁No

3.3. Are you willing to offer opportunistic CT screening in clinical practice?

➀Yes ➁No

1. **Training and Chlamydia trachomatis testing experience**

4.1. Have you ever attended to any training on STIs diagnosis and treatment?

➀Yes ➁No

4.2. Have you been involved in Chlamydia trachomatis testing in last 3 months?

➀Yes ➁No (skip to section 5.)

4.3. How many Chlamydia trachomatis tests did you perform per month?

➀ 1-5; ➁ 6-10 ➂ more than 10.

4.4. What type of Chlamydia trachomatis testing would you choose as your first choice to detect Chlamydia trachomatis infection? ___________________

4.5. For men who have sex with men, what kind of specimen is recommended to collect? ___________________

1. **Chlamydia trachomatis practice**

5.1. Are you willing to remind Chlamydia trachomatis positive patients of partner notification?

➀Yes ➁No

5.2. What is the appropriate time frame of the Chlamydia trachomatis retesting?

➀ 2 weeks; ➁ 8 weeks; ➂ 3 months

5.3. What type of medicine would you choose as the first choice to treat men or non-pregnant women with uncomplicated chlamydia infection? ___________________

5.4. What type of medicine would you choose as the first choice to treat pregnant women with uncomplicated chlamydia infection? ___________________
